# 1.5 versus 3 versus 7 Tesla in abdominal MRI: A comparative study

**DOI:** 10.1371/journal.pone.0187528

**Published:** 2017-11-10

**Authors:** Anja Laader, Karsten Beiderwellen, Oliver Kraff, Stefan Maderwald, Karsten Wrede, Mark E. Ladd, Thomas C. Lauenstein, Michael Forsting, Harald H. Quick, Kai Nassenstein, Lale Umutlu

**Affiliations:** 1 Department of Diagnostic and Interventional Radiology and Neuroradiology, University Hospital Essen, Hufelandstr. 55, Essen, Germany; 2 Erwin L. Hahn Institute for Magnetic Resonance Imaging, University Duisburg-Essen, Kokereiallee 7, Essen, Germany; 3 Department of Neurosurgery, University Hospital Essen, Hufelandstr. 55, Essen, Germany; 4 German Cancer Research Center (DKFZ), Im Neuenheimer Feld 280, Heidelberg, Germany; 5 Institute of Radiology, Evangelisches Krankenhaus Düsseldorf, Kirchfeldstr. 40, Düsseldorf, Germany; 6 High Field and Hybrid MR Imaging, University Hospital Essen, Hufelandstr. 55, Essen, Germany; Universitair Medisch Centrum Utrecht, NETHERLANDS

## Abstract

**Objectives:**

The aim of this study was to investigate and compare the feasibility as well as potential impact of altered magnetic field properties on image quality and potential artifacts of 1.5 Tesla, 3 Tesla and 7 Tesla non-enhanced abdominal MRI.

**Materials and methods:**

Magnetic Resonance (MR) imaging of the upper abdomen was performed in 10 healthy volunteers on a 1.5 Tesla, a 3 Tesla and a 7 Tesla MR system. The study protocol comprised a (1) T1-weighted fat-saturated spoiled gradient-echo sequence (2D FLASH), (2) T1-weighted fat-saturated volumetric interpolated breath hold examination sequence (3D VIBE), (3) T1-weighted 2D in and opposed phase sequence, (4) True fast imaging with steady-state precession sequence (TrueFISP) and (5) T2-weighted turbo spin-echo (TSE) sequence. For comparison reasons field of view and acquisition times were kept comparable for each correlating sequence at all three field strengths, while trying to achieve the highest possible spatial resolution. Qualitative and quantitative analyses were tested for significant differences.

**Results:**

While 1.5 and 3 Tesla MRI revealed comparable results in all assessed features and sequences, 7 Tesla MRI yielded considerable differences in T1 and T2 weighted imaging. Benefits of 7 Tesla MRI encompassed an increased higher spatial resolution and a non-enhanced hyperintense vessel signal at 7 Tesla, potentially offering a more accurate diagnosis of abdominal parenchymatous and vasculature disease. 7 Tesla MRI was also shown to be more impaired by artifacts, including residual B_1_ inhomogeneities, susceptibility and chemical shift artifacts, resulting in reduced overall image quality and overall image impairment ratings. While 1.5 and 3 Tesla T2w imaging showed equivalently high image quality, 7 Tesla revealed strong impairments in its diagnostic value.

**Conclusions:**

Our results demonstrate the feasibility and overall comparable imaging ability of T1-weighted 7 Tesla abdominal MRI towards 3 Tesla and 1.5 Tesla MRI, yielding a promising diagnostic potential for non-enhanced Magnetic Resonance Angiography (MRA). 1.5 Tesla and 3 Tesla offer comparably high-quality T2w imaging, showing superior diagnostic quality over 7 Tesla MRI.

## Introduction

The increase of the magnetic field strength goes hand in hand with changes in physical features such as signal-to-noise-ratio (SNR), tissue susceptibility, chemical shift, or radiofrequency (RF) effects, yielding potentially beneficial as well as disadvantageous effects [1,2]. While the urge to increase the magnetic field strength is primarily driven by the associated increase in SNR, that can be traded off into faster image acquisition and/or improved spatial resolution, high-field MRI can also be impaired due to exacerbation of artifacts and specific absorption rate (SAR) limitations. Starting out with imaging neuroradiological and musculoskeletal MRI, various studies demonstrated the feasibility as well as the potential diagnostic superiority of ultra-high-field MRI [3–8]. The increase of the associated SNR could be successfully transitioned into imaging at higher spatial resolution, allowing for improved assessment of anatomical and pathological structures [4,8–12]. Continuing developments in multi-channel transmit/receive RF body coil technology and B_1_ shimming allowed to uplift body imaging to higher field strengths [1,12–18]. 3 Tesla abdominal MRI has been progressively established in clinical diagnostics, providing diagnostic equivalence in specific applications, demanding for fast repetitive image acquisition at high spatial resolution to perform dynamic liver MRI or renal MR angiography [19–22]. Initial studies of ultra-high-field abdominal MRI have revealed the feasibility and diagnostic potential of liver and kidney MRI as well as MRA at 7 Tesla [23–26]. Nevertheless, the increase of the field strength to 7 Tesla did not only provide beneficial aspects in abdominal MRI but also yielded significant challenges and impairments due to an exacerbation of artifacts and limitations in SAR.

With the benefits of increasing field strength for neuroradiological and musculoskeletal applications and the successful implementation of 3 Tesla and 7 Tesla MR imaging to abdominal imaging, the aim of this study was to intraindividually investigate and compare the potential impact of altered magnetic field properties on image quality, image contrast and potential artifacts of 7 Tesla compared to 3 Tesla and 1.5 Tesla non-enhanced abdominal MRI.

## Materials and methods

### Study population

Ten healthy volunteers (six male and four female subjects; average age: 29.5 years, range 26–33 years) were enrolled in this trial. The mean body mass index (BMI) was 24.07 ± 1.83 (range 20.96–27.94). The study was conducted in conformance with the Declaration of Helsinki and approved by the Ethics Commission of the Medical Faculty of the University Duisburg-Essen (study number 11-4898-BO). Written informed consent was obtained from each volunteer before the examination.

### 1.5 Tesla MR scanner and RF coil system

Examinations were performed on a 1.5 Tesla whole-body MR system (Magnetom Aera, Siemens Healthcare Sector, Erlangen, Germany) The gradient system enables a maximum amplitude of 45 mT/m and a slew rate of 220 mT/m/ms. For image acquisition an 18-channel body flex coil (Siemens, Siemens Healthcare Sector, Erlangen, Germany) was applied.

### 3 Tesla MR scanner and RF coil system

High-field examinations were obtained on a 3 Tesla whole-body MR system (Magnetom Skyra, Siemens Healthcare Sector, Erlangen, Germany) with a gradient system enabling a maximum amplitude of 45 mT/m and a slew rate of 220 mT/m/ms. For image acquisition an 18-channel body flex coil (Siemens, Siemens Healthcare Sector, Erlangen, Germany) was applied.

### 7 Tesla MR scanner and RF coil system

Ultra-high field examinations were performed on a 7 Tesla whole-body MR system (Magnetom 7T, Siemens Healthcare Sector, Erlangen, Germany). The scanner is equipped with a gradient system enabling 38 mT/m maximum amplitude and a slew rate of 200 mT/m/ms.

Due to the unavailability of commercially procurable coils for ultra-high-field abdominal MRI, a custom-built 8-channel body transmit receive coil was applied for image acquisition at 7 Tesla. The coil system is composed of a set of symmetrically excited meander strip line elements to provide best possible intrinsic decoupling and a larger penetration depth in the imaged subject. The coil is constructed of two arrays, with four elements each, placed on the ventral and dorsal upper half of the abdomen. The elements of the ventral array are arranged in individual modules of Macrolon, interconnected with a Neoprene sheet, to facilitate flexibility to the shape of the individual subject´s anatomy and ensure best possible B_1_^+^ uniformity. To ensure flexibility along the longitudinal axis of the subject, the elements of the dorsal array are embedded in a plane on a sliding frame [27].

**Fig 1:**
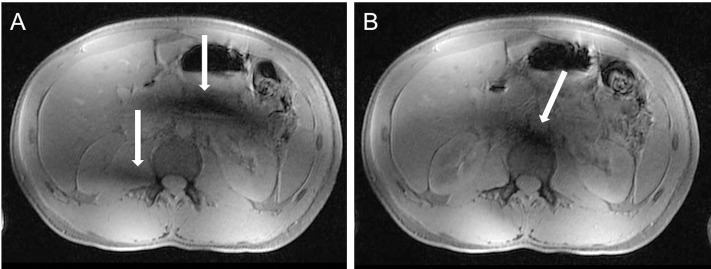
RF shimming. 7 Tesla 3D VIBE imaging using the CP (Figure A) and CP^2+^ mode (Figure B) to shift and focus B_1_ inhomogeneities from the right liver lobe and peripancreatic area (arrows Figure A) to a less disturbing focal periaortal area (arrow Figure B).

As ultra-high-field MR systems do not offer automatic B_0_ and B_1_ shimming at current status, manual B_0_ shimming was performed using a vendor-provided gradient echo sequence and algorithm prior to the acquisition of diagnostic sequences. The increase of the magnetic field strength is associated to a reduced Larmor wavelength, resulting in B_1_ field inhomogeneities caused by destructive B_1_ interference. To obtain best possible homogeneity, a custom-built 8-channel add-on system for RF-shimming was integrated on the small-signal side of the MR system including vector modulation on each transmit channel [28]. After hardware and software modification, RF-shimming could be performed by splitting the excitation signal of the conventional single-channel system into 8 independent channels. Hence, the application of optimized sets of amplitude and phase shifts to obtain a uniform excitation of specific body regions was enabled. For transversal imaging a second-order circularly polarized mode (CP2+; equal amplitudes and a phase increment of 90° between neighboring elements) transitioning potential signal voids to a small periaortal focus was applied in all acquired sequences being beneficial compared to the first-order birdcage mode (CP mode) with a phase increment of 45° between neighboring elements and equal amplitudes, shifting potential signal voids to the right upper quadrant of the abdomen (Fig 1).

For safety reasons, real-time RF supervision was performed based on logarithmic power meters monitoring forward and reflected power. A real-time field programmable gate array (NI PXI 7852R, National Instruments, Austin, Texas, USA) offered time-averaging of the transmitted RF power according to IEC safety guidelines [29]. To stay within SAR regulations, average power limits were calculated from SAR simulations based on body models from the Visible Human Project (CST Microwave Studio, CST AG, Darmstadt, Germany) and the Virtual Family [30].

### Examination protocol and modification of parameters

Imaging parameters [repetition time (TR), echo time (TE), flip angle and bandwidth] were modified to compensate for changes in physical features affiliated to the increase of the magnetic field strength. For comparison reasons field of view and acquisition times were kept comparable for each correlating sequence at all three field strengths, while trying to achieve the highest possible spatial resolution. The examination protocol included the following sequences:

1. T1-weighted fat-saturated spoiled gradient-echo sequence (2D FLASH)
2. T1-weighted fat-saturated volumetric interpolated breath hold examination sequence (3D VIBE)
3. T1-weighted 2D in and opposed phase sequence
4. True fast imaging with steady-state precession sequence (TrueFISP)
5. T2-weighted turbo spin-echo (TSE) sequence

Detailed information on imaging parameters is are summarized in Tables 1 and 2. Sequence parameters were adapted at the different field strengths to obtain best image quality, highest exploitation of SNR, and with regard to workflow and SAR restrictions. At 7 Tesla, a slightly smaller base resolution of 448 was chosen while allowing 100% resolution in phase direction, compared to 512 and 50% at 3 Tesla. In addition, the receiver bandwidth at 7 Tesla was doubled compared to 3 Tesla to counteract the increase in fat-water shift. Both, increase in bandwidth and decrease in base resolution led to a smaller TR/TE combination at 7 Tesla compared to 3 Tesla. For the T2 weighted TSE sequence, for example, SAR limitations forced to choose lower refocusing pulses at 7 Tesla compared to 1.5 Tesla and 3 Tesla despite the consequence of having a less effective refocusing and an increase in artifacts arising from stimulated echoes. SAR limitations and lack of suitable B_1_ mapping techniques for large cross-sections at 7 Tesla on one hand, as well as limited available peak RF power on the other hand [31] forced to use a fixed transmitter voltage for all subjects at 7 Tesla. At lower field strengths, the MR system of course determined the RF transmitter voltage individually through automatic adjustments.

**Table 1:** Detailed imaging parameters for the T1 weighted sequences.

	Slice orientation	Fat saturation	Repetition time TR [ms]	Echo time TE [ms]	Field-of-view FOV [mm^2^]	Acquistion matrix [pixel]
**2D FLASH**						
1.5 Tesla	axial	Yes	162	2.34	350x263	288x230
3 Tesla	axial	Yes	234	2.8	350x263	320x224
7 Tesla	axial	Yes	193	3.57	350x263	448x448
**3D VIBE**						
1.5 Tesla	axial	Yes	3.52	1.54	350x263	512x256
3 Tesla	axial	Yes	4.72	2.32	350x263	512x256
7 Tesla	axial	Yes	3.2	1.26	350x228	448x448
**In-opp. Phase**						
1.5 Tesla	axial	None	124	2.22	350x263	256x192
3 Tesla	axial	None	174	1.29	350x263	288x288
7 Tesla	axial	None	232	2.04/3.57	350x263	320x320
	**Voxel volume [mm^3^] n.i.**	**Slices**	**Parallel acquisition with GRAPPA**	**Flip angle [°]**	**Bandwidth** **[Hz/pixel]**	**Acq. Time [min]**
**2D FLASH**						
1.5 Tesla	1.2x.1.5x7.0	30	2(24)	70	290	0:46
3 Tesla	1.1x1.6x7.0	30	2(24)	70	210	0:50
7 Tesla	0.8x0.8x5.0	42	2(48)	70	410	0:52
**3D VIBE**						
1.5 Tesla	0.7x1.4x6.0	80	none	12	470	0:21
3 Tesla	0.7x1.3x6.0	80	2(24)	12	440	0:18
7 Tesla	0.8x0.8x3.0	120	2(48)	10	930	0:24
**In-opp. Phase**						
1.5 Tesla	1.4x1.8x7.0	30	2(24)	70	390	0:21
3 Tesla	1.2x1.2x7.0	30	2(32)	70	1090	0:22
7 Tesla	1.1x1.1x6.0	42	3(50)	65	980	0:22

**Table 2:** Detailed imaging parameters for the T2w TSE sequence and TrueFISP imaging.

	Slice orientation	Fat saturation	Repetition time TR [ms]	Echo time TE [ms]	Field-of-view FOV [mm^2^]	Acquistion matrix [pixel]
**T2 TSE**						
1.5 Tesla	axial	None	3900	83	350x263	256x256
3 Tesla	axial	None	3960	96	350x263	320x224
7 Tesla	axial	None	3500	90	350x263	320x224
**TrueFISP**						
1.5 Tesla	coronal	None	3.50	1.37	350x350	320x240
3 Tesla	coronal	None	3.69	1.61	350x350	320x224
7 Tesla	coronal	None	4.37	2.19	350x350	384x384
	**Voxel volume [mm^3^] n.i.**	**Slices**	**Parallel acquisition with GRAPPA**	**Flip angle** **[°]**	**Bandwidth** **[Hz/pixel]**	**Acq.** **time** **[min]**
**T2 TSE**						
1.5 Tesla	1.4x1.7x7.0	30	2(78)	180	260	0:44
3 Tesla	1.1x1.5x7.0	30	2(30)	160	260	0:50
7 Tesla	1.1x1.5x7.0	30	2(48)	150	244	0:49
**TrueFISP**						
1.5 Tesla	1.1x1.5x5.0	38	2(24)	66	504	0:22
3 Tesla	1.1x1.1x5.0	28	2(24)	70	919	0:24
7 Tesla	0.9x0.9x3.0	42	2(24)	70	1085	0:28

After the examinations, all subjects were interviewed about side effects and acceptance with regards to the examination time, positioning, and sensations such as vertigo, dizziness or nausea.

### Image analysis and statistical evaluation

Visual evaluation was performed on a standard post-processing Picture Archiving and Communication System (PACS) workstation (Centricity RIS 4.0i, GE Healthcare, USA) by two senior radiologists with 12 and 9 years expertise in abdominal MRI in consensus.

Qualitative image analysis was assessed utilizing a five-point-scale with regard to overall contrast resolution, sharpness and clarity: 5 = excellent quality (very high contrast resolution and signal homogeneity, high sharpness and clarity of vessels, organs and background signal), 4 = good quality (slightly reduced contrast resolution and signal homogeneity, good sharpness and clarity of vessels, organs and background signal), 3 = moderate quality (moderate contrast resolution or signal homogeneity, fair sharpness and clarity), 2 = poor quality (clearly reduced but sufficient contrast resolution and signal homogeneity, poor sharpness and clarity), 1 = non-diagnostic (considerably reduced contrast resolution and signal homogeneity, low sharpness and clarity). The overall image impairment due to artifacts including chemical shift, B_1_ inhomogeneities, susceptibility and motion artifacts was assessed using the following 5-point scale: 5 = no impairment, 4 = slight impairment or insignificant, 3 = moderate impairment, 2 = strong impairment, 1 = non-diagnostic due to artifact. Furthermore, T1-weighted sequences (2D FLASH, 3D VIBE and in- and opposed phase imaging) were dedicatedly assessed for the delineation of non-enhanced arterial vasculature: 5 = excellent sharpness and homogeneous delineation, 4 = good sharpness and slightly inhomogeneous delineation, 3 = moderate sharpness and inhomogeneous delineation, 2 = poor sharpness and inhomogeneous delineation, 1 = not visible. Additionally, TrueFISP imaging and T2-weighted TSE MRI were evaluated for the delineation of the biliary duct system: 5 = excellent and sharp delineation, 4 = good delineation, 3 = moderate delineation, 2 = poor delineation, 1 = not visible.

For quantitative evaluation, contrast ratios (CR) were calculated (CR = [signal_tissue_−signal_psoas_] / [signal_tissue_ + signal_psoas_]), similar to previous comparison studies [21]. Signal was measured by placing regions of interest (ROIs) of identical size into the organ tissue and ipsilateral psoas muscle as follows: (1) right liver lobe (segment 6) and right psoas muscle, (2) left liver lobe (segment 3) and left psoas muscle, (3) spleen and left psoas muscle and (4) left renal cortex and left psoas muscle. Qualitative and quantitative results are displayed as mean values. In accordance with previous comparison trials on different field strength publications [32], no measurements of SNR and CNR were performed as noise shows an inhomogeneous distribution in images acquired with parallel imaging, thus strongly impairing noise measurements [33].

For statistical analysis, a Wilcoxon rank test was used. Scoring values of image quality and presence of artifacts for each sequence were compared. For every mean value the standard error of mean (SE = σ / √n) was calculated as an estimate of the population mean. To account for multiple testing a p-value < 0.01 was considered to represent statistically significant differences. Statistical analysis was carried out with the STATA software package (Stata/SE 12.1 for Mac (64-bit Intel), StataCorp, 4905 Lakeway Drive, College Station, Texas 77845 USA).

## Results

All MR examinations were successfully obtained and were well tolerated without significant side effects. The mean examination time for the 1.5 Tesla and 3 Tesla scans amounted to 24 minutes (+/- 5 minutes) each. The mean examination time for the 7 Tesla examination amounted to 35 (+/- 5 minutes), as ultra-high-field imaging requires an additional manual 3D B_0_ shim and slow positioning of the subjects into the bore.

With increasing magnetic field strength, all T1-weighted sequences could be obtained with higher spatial resolution at equivalent acquisition times. While overall image quality was rated comparably high for T1w imaging in all three field strengths, 7 Tesla MRI demonstrated its superiority in the assessment of non-enhanced arterial vasculature based on a homogeneous hyperintense vessel signal. T2-weighted MRI provided equally high quality imaging in 1.5 and 3 Tesla, while 7 Tesla T2-weighted TSE MRI showed significantly reduced image quality, also reflected in significantly stronger overall image impairment. Quantitative assessment revealed highest contrast ratios at 7 Tesla for all analyzed organ tissues in all T1-weighted sequences, while 1.5 Tesla and 3 Tesla showed higher values for T2-weighted sequences.

**Fig 2:**
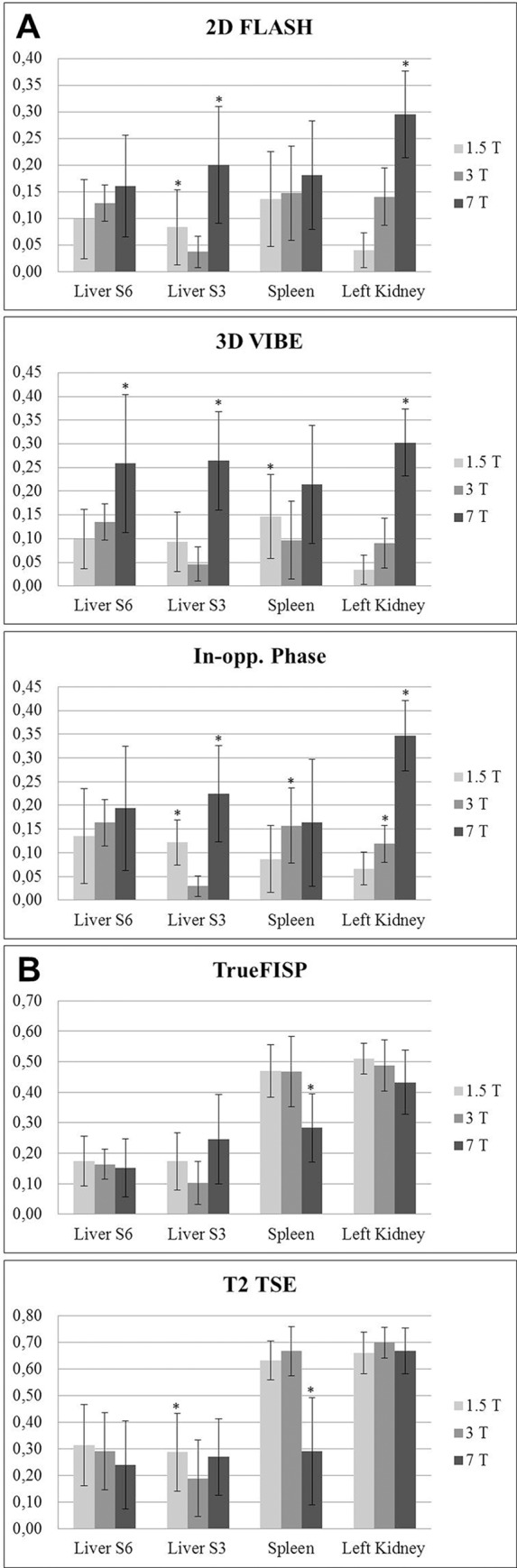
Quantitative evaluation. (A) Quantitative analysis showing contrast ratios of different parenchymatous organs to the ipsilateral psoas muscle for the T1-weighted sequences. * indicates statistical significance (p value < 0.05).(B) Quantitative analysis showing contrast ratios of different parenchymatous organs to the ipsilateral psoas muscle for TrueFISP and T2-weighted TSE sequence. * indicates statistical significance (p value < 0.05).

All results for image quality and presence of artifacts including mean values and standard deviation are displayed in Tables 3 and 4 Results for quantitative evaluation are showed in Fig 2. Detailed results for each sequence are listed below.

**Table 3:** Qualitative image evaluation.

	Overall image quality	Overall image impairment	Chemical shift	B_1_ inhomogeneity
**2D FLASH**				
1.5 Tesla	4.4 +/- 0.8	4.6 +/- 0.49	4.8 +/- 0.4	4.9 +/- 0.04
3 Tesla	4.6 +/- 0.55	4.4 +/- 0.55	4.9 +/- 0.18	4.9 +/- 0.17
7 Tesla	4.2 +/- 0.79	3.8 +/- 0.43^#^	4.0 +/- 0.33^#^*	3.6 +/- 0.52^#^*
**3D VIBE**				
1.5 Tesla	4.6 +/- 0.55	4.8 +/- 0.45	4.6 +/- 0.55	4.5 +/- 0.52
3 Tesla	4.6 +/- 0.43	4.7 +/- 0.40	4.6 +/- 0.50	4.4 +/- 0.46
7 Tesla	4.2 +/- 0.79	4.4 +/- 0.72^#^*	4.3 +/- 0.41^#^*	4.2 +/- 0.65^#^*
**In-opp. Phase**				
1.5 Tesla	4.6 +/- 0.55	4.6 +/- 0.55	4.5 +/- 0.89	4.8 +/- 0.45
3 Tesla	4.5 +/- 0.55	4.4 +/- 0.55	4.2 +/- 0.75	4.5 +/- 0.55
7 Tesla	4.3 +/- 0.52	4.2 +/- 0.75	3.7 +/- 0.52^#^	3.7 +/- 0.54^#^*
	**Susceptibility**	**Motion artifact**	**Vessel delineation**	
**2D FLASH**				
1.5 Tesla	4.9 +/- 0.08	4.3 +/- 0.43	1.9 +/- 0.08	
3 Tesla	4.9 +/- 0.11	4.6 +/- 0.42	1.8 +/- 0.34	
7 Tesla	4.2 +/- 0.83^#^*	4.3 +/- 0.61*	4.2 +/- 0.49^#^*	
**3D VIBE**				
1.5 Tesla	3.8 +/- 0.84	4.6 +/- 0.42	1.9 +/- 0.18	
3 Tesla	3.9 +/- 0.73	4.3 +/- 0.83	1.8 +/- 0.17	
7 Tesla	3.6 +/- 0.41	4.1 +/- 0.53	3.7 +/- 0.32^#^*	
**In-opp. Phase**				
1.5 Tesla	4.4 +/- 0.89	4.4 +/- 0.89	1.9 +/- 0.71	
3 Tesla	4.6 +/- 0.56	4.2 +/- 0.75	2.1 +/- 0.63	
7 Tesla	4.2 +/- 0.75*	4.0 +/- 0.63	3.8 +/- 0.41^#^*	

Mean values and standard deviation of qualitative image evaluation of T1-weighted sequences: 2D FLASH, 3D VIBE, in- and-opposed phase imaging); (5 = excellent quality, 4 = good quality, 3 = moderate quality, 2 = poor, 1 = non-diagnostic). Significant differences between 1.5 Tesla and 7 Tesla are marked with ^#^. Significant differences between 3 Tesla and 7 Tesla are marked with *.

**Table 4:** Qualitative image evaluation.

	Overall image quality	Overall image impairment	Chemical shift	B_1_ inhomogeneity
**TrueFISP**				
1.5 Tesla	4.4 +/- 0.55	4.4 +/- 0.55	4.2 +/- 0.84	4.2 +/- 0.45
3 Tesla	4.2 +/- 0.44	4.4 +/- 0.54	4.0 +/- 0.70	4.4 +/- 0.55
7 Tesla	3.4 +/- 0.55^#^*	3.6 +/- 0.55^#^*	3.7 +/- 0.54^#^*	3.0 +/- 0.71^#^*
**T2 TSE**				
1.5 Tesla	4.8 +/- 0.45	4.6 +/- 0.55	4.9 +/- 0.09	4.8 +/- 0.45
3 Tesla	4.9 +/- 0.09	4.8 +/- 0.45	4.9 +/- 0.13	4.9 +/- 0.04
7 Tesla	2.6 +/- 0.55^#^*	2.0 +/- 0.71^#^*	4.2 +/- 0.45	3.4 +/- 0.55^#^*
	**Susceptibility**	**Motion artifact**	**Biliary duct system**	
**TrueFISP**				
1.5 Tesla	4.2 +/- 0.84	4.8 +/- 0.45	4.6 +/- 0.55	
3 Tesla	4.2 +/- 0.45	4.8 +/- 0.45	4.5 +/- 0.71	
7 Tesla	3.8 +/- 0.45*	4.6 +/- 0.55	3.2 +/- 0.45^#^	
**T2 TSE**				
1.5 Tesla	4.8 +/- 0.45	4.4 +/- 0.55	4.9 +/- 0.09	
3 Tesla	4.6 +/- 0.55	4.6 +/- 0.56	4.9 +/- 0.04	
7 Tesla	3.8 +/- 0.45^#^*	3.4 +/- 0.89	1.4 +/- 0.55^#^*	

Mean values and standard deviation of qualitative image evaluation of T2-weighted TSE imaging and TrueFISP imaging (5 = excellent quality, 4 = good quality, 3 = moderate quality, 2 = poor, 1 = non-diagnostic). Significant differences between 1.5 Tesla and 7 Tesla are marked with ^#^. Significant differences between 3 Tesla and 7 Tesla are marked with *.

### T1-weighted spoiled gradient-echo sequence (2D FLASH)

**Fig 3:**
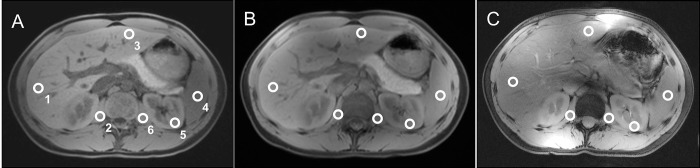
2D FLASH. 2D FLASH imaging at 1.5 Tesla (A), 3 Tesla (B) and 7 Tesla (C) in the same subject. The white circles indicate the ROIs for signal measurements in the right liver lobe (1), right psoas muscle (2), left liver lobe (3), spleen (4), left kidney cortex (5) and left psoas muscle (6). Note the slight motion artifacts due to breathing as well as the slight loss in contrast at 3 Tesla (B) compared to 1.5 Tesla (A) and the hyperintense delineation of intra-abdominal vasculature at 7 Tesla (C).

Qualitative image analysis revealed similar score values for overall image quality in all three field strengths, ranging from mean 4.2 for 7 Tesla MRI to mean 4.6 for 3 Tesla MRI, with a significant difference between 7 Tesla and 3 Tesla (p<0.01). While 1.5 and 3 Tesla showed comparable artifact delineation, 7 Tesla MRI revealed significantly higher impairment due to chemical shift, B_1_ inhomogeneity and susceptibility artifacts, resulting in lower overall image impairment ratings (3.8_7Tesla_, 4.4_3Tesla_, 4.6_1.5Tesla_). 7 Tesla MRI provided significantly higher ratings for vessel delineation compared to the lower field strengths (4.2_7Tesla_, 1.8_3Tesla_, 1.8_1.5Tesla;_ p<0.004). An example for intraindividual 2D FLASH imaging at all three field strengths as well as placement of ROIs for quantitative evaluation is displayed in Fig 3.

Quantitative assessment of 2D FLASH revealed highest contrast ratio values for liver, spleen and left kidney tissue (to psoas muscle) at 7 Tesla when compared to 3 Tesla and 1.5 Tesla, yielding significantly higher values for the left liver lobe (CR_7Tesla_ 0.20, CR_3Tesla_ 0.04, CR_1.5Tesla_ 0.08) with p<0.035 (7 Tesla vs. 3 Tesla) and p<0.012 (7 Tesla vs. 1.5 Tesla) and for the left kidney (CR_7Tesla_ 0.30, CR_3Tesla_ 0.14, CR_1.5Tesla_ 0.04) with p<0.012 respectively.

### T1-weighted spoiled gradient-echo sequence (3D VIBE)

**Fig 4:**
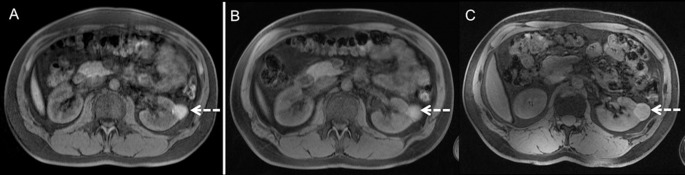
3D VIBE. 3D VIBE imaging at 1.5 Tesla (A), 3 Tesla (B) and 7 Tesla (C) in the same subject. Note equivalent overall image quality, contrast, homogeneous fat saturation and signal homogeneity at all three field strengths. Dashed arrows point at incidental hemorrhaged renal cyst.

Overall image quality, in terms of overall contrast resolution, sharpness and clarity was rated comparably high for all three magnetic field strengths, with only insignificantly lower values for 7 Tesla imaging (4.2_7Tesla_, 4.6_3Tesla_, 4.6_1.5Tesla_). The analysis of overall image impairment revealed significantly decreased scoring for 7 Tesla MRI (4.4_7Tesla_, 4.7_3Tesla_, 4.8_1.5Tesla_), mainly based on residual B_1_ inhomogeneities despite best possible RF shimming. Despite the lack of intravenous contrast agent, the inherently high signal intensity of the arterial vasculature in 7 Tesla T1w MRI provided high-quality conspicuity of the intraabdominal vessels (3.7_7Tesla_, 1.8_3Tesla_, 1.9_1.5Tesla_) (Fig 4).

7 Tesla showed highest CR values for all analyzed organs compared to 3 Tesla and 1.5 Tesla with highest values for the left kidney (CR_7Tesla_ 0.30, CR_3Tesla_ 0.09, CR_1.5Tesla_ 0.03) with p<0.012 respectively. CR at 7 Tesla are significantly higher than 3 Tesla and 1.5 Tesla for the right and left liver lobe as well as the left kidney (p from 0.012–0.018), and not-significantly higher for the spleen (p 0.128–0.484). Despite highest values at 7 Tesla, 1.5 Tesla showed higher contrast ratios than 3 Tesla for the left liver lobe S3 (CR_7Tesla_ 0.26, CR_3Tesla_ 0.05, CR_1.5Tesla_ 0.09) with p<0.068 and the spleen (CR_7Tesla_ 0.21, CR_3Tesla_ 0.10, CR_1.5Tesla_ 0.15), p<0.034.

### T1-weighted 2D in and opposed phase sequence

**Fig 5:**
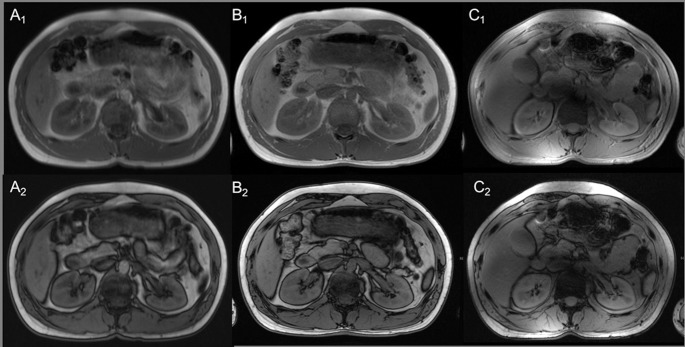
T1-weighted 2D in and opposed phase. T1-weighted 2D in and opposed phase imaging at 1.5 Tesla (A_1,2_) 3 Tesla (B_1,2_) and 7 Tesla (C_1,2_) in the same subject. The typical black rims with a fat/water interface around organs are increasingly sharp and clear visualized in the opposed phase images at 3 and 7 Tesla, compared to 1.5 Tesla and are also visible in the in phase images at 7 Tesla. Nevertheless, strong signal voids due to RF shimming are detectable in the periaortal region at 7 Tesla images. The expected exacerbation of chemical shifting (3.7_7Tesla_, 4.4_3Tesla_, 4.5_1.5Tesla_) associated to the increase of the magnetic field strength resulted in insignificantly decreased mean values of overall image quality and impairment at 7 Tesla compared to 1.5 and 3 Tesla (4.2_7Tesla_, 4.4_3Tesla_, 4.6_1.5Tesla_). Additionally 7 Tesla MRI was relatively strongly impaired due to residual B_1_ inhomogeneities (3.7_7Tesla_, 4.5_3Tesla_, 4.8_1.5Tesla_). Being a T1w gradient echo sequence, in and opposed phase imaging also provided moderate to high-quality delineation of the non-enhanced vasculature at 7 Tesla (3.8_7Tesla_, 2.1_3Tesla_, 1.9_1.5Tesla_). In accordance to the previous T1w sequences, 7 Tesla demonstrated higher contrast ratios compared to 3 Tesla and 1.5 Tesla for all organs with highest difference for the left kidney (CR_7Tesla_ 0.35, CR_3Tesla_ 0.12, CR_1.5Tesla_ 0.07), p<0.012 respectively. For the right liver lobe S6 (CR_7Tesla_ 0.19, CR_3Tesla_ 0.16, CR_1.5Tesla_ 0.14) and the spleen (CR_7Tesla_ 0.17, CR_3Tesla_ 0.16, CR_1.5Tesla_ 0.09), 7 Tesla revealed slightly higher values without statistical significance (p values from 0.326–1).

Out-of-phase imaging could be obtained with the typical sharply defined black rims around organs with a fat/water interface in all three magnetic fields (Fig 5).

### True fast imaging with steady-state precession (True-FISP)

Being a sequence with strong susceptibility to B_0_ field inhomogeneity, TrueFISP imaging was the sequence to suffer strongest from enhanced banding artifacts, when comparing 7 Tesla to lower field strengths. Consequently, overall image quality (3.4_7Tesla_, 4.2_3Tesla_, 4.4_1.5Tesla_) and overall image impairment (3.6_7Tesla_, 4.4_3Tesla_, 4.4_1.5Tesla_) were markedly reduced at 7 Tesla over 1.5 and 3 Tesla. The analysis of the biliary duct system revealed comparably high delineation at 1.5 and 3 Tesla and only moderate conspicuity at 7 Tesla (3.2_7Tesla_, 4.5_3Tesla_, 4.6_1.5Tesla_). Evaluation of contrast ratios for TrueFISP showed decreasing CR values with increasing field strength with 7 Tesla showing significantly lower values for the spleen tissue (CR_7Tesla_ 0.28, CR_3Tesla_ 0.47, CR_1.5Tesla_ 0.47) with p<0.012 and non-significantly lower values for the right liver lobe S6 and left kidney.

### T2-weighted turbo spin-echo sequence

**Fig 6:**
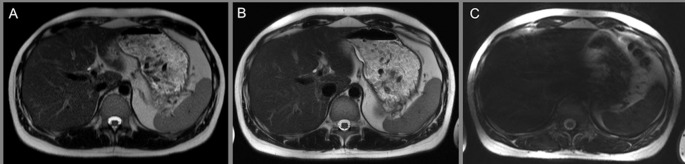
T2 TSE. T2 TSE imaging at 1.5 Tesla (A_1,2_) 3 Tesla (B_1,2_) and 7 Tesla (C_1,2_) in the same subject. Note equivalent image quality and contrast at 1.5 Tesla (A) and 3 Tesla (B). Despite the increased spatial resolution no improvement can be detected at 3 Tesla (B). Figure C shows strongly impaired T2 TSE imaging at 7 Tesla, revealing strong signal loss in parenchymatous organs. Hence, assessment of the biliary duct system was significantly hampered at 7 Tesla field strength (1.4_7Tesla_, 4.9_3Tesla_, 4.9_1.5Tesla_). Quantitative evaluation confirmed limitations of T2w TSE at 7 Tesla showing lowest CR values for all analyzed organs compared to 3 Tesla and 1.5 Tesla, being significantly lower for the spleen tissue (CR_7Tesla_ 0.29, CR_3Tesla_ 0.67, CR_1.5Tesla_ 0.63) with p<0.012, and non-significantly lower for liver and kidney tissue. Detailed graphs for evaluation of contrast ratios are displayed in Fig 2.

1.5 Tesla and 3 Tesla T2w TSE imaging showed comparably excellent score values in all assessed parameters with only minor impairment based on motion artifacts. Further increase of the field strength to 7 Tesla revealed limitations of TSE imaging with strictly decreased ratings of overall image quality (2.6_7Tesla_, 4.9_3Tesla_, 4.8_1.5Tesla_), as well as overall and dedicated image impairment analysis (2.0_7Tesla_, 4.8_3Tesla_, 4.6_1.5Tesla_), (Fig 6).

## Discussion

Recent developments in multi-channel transmit/receive RF body coil technology and B_1_ shimming have enabled a platform for the successful implementation of high-field (3 Tesla) and ultra-high-field (7 Tesla) MRI to abdominal imaging. With 1.5 Tesla MRI remaining to be the worldwide clinical standard, 3 Tesla abdominal MRI has demonstrated its potential as an equivalent and in particular applications even superior diagnostic imaging technique [19–21]. Falkenhausen et al. published initial results on 3 Tesla liver MRI, demonstrating the diagnostic equivalence to 1.5 Tesla MRI in terms of diagnostic accuracy regarding the detection and classification of focal liver lesions [21]. Nevertheless, other publications also stressed the increased artifact delineation and associated image impairment when comparing high-resolution whole-body MRI at 3 Tesla with 1.5 Tesla [32]. Lately, body MRI at ultra-high-field strength, by means of 7 Tesla MRI, has been introduced for in vivo application in humans. Starting out with the acquisition of landscaping image sets to demonstrate the general feasibility and safety of 7 Tesla body and cardiac MRI [34,35] more recent publications have focused on the implementation of standardized imaging protocols to investigate the diagnostic potential of 7 Tesla abdominal MRI, focussing on dedicated non-enhanced as well as contrast-enhanced liver and kidney imaging as well as renal MR angiographic applications [24–26]. Qualitative image analysis revealed high-quality T1-weighted MRI with excellent delineation of anatomical details and minor impairment due to artifacts based on successful shimming techniques. Nevertheless, the trials also revealed considerable differences between T1w and T2w imaging with regard to overall image quality and image impairment due to artifacts, as T2-weighted MRI showed significant restraints based on the exacerbation of artifacts.

To our knowledge the current trial is the first study to provide an intraindividual comparison of abdominal MR imaging at all three magnetic field strengths. Our results confirm published results, in terms of the diagnostic equivalence of 1.5 and 3 Tesla abdominal MRI [19–22] and underline the strengths and drawbacks of 7 Tesla T1-weighted abdominal MR imaging [17, 31]. The increase of the magnetic field strength from 1.5 Tesla to 3 Tesla allowed for imaging at higher spatial resolution for all T1w and T2w sequences, while maintaining equal acquisition time and FOV (Tables 1 and 2). Nevertheless no significant differences were detected based on the increased spatial resolution, as qualitative analysis of overall image quality and detailed as well as overall artifact impairment revealed comparable results for all assessed sequences. The expected exacerbation of artifacts, such as chemical shifting, did not lead to a relevant impairment of image quality.

**Fig 7:**
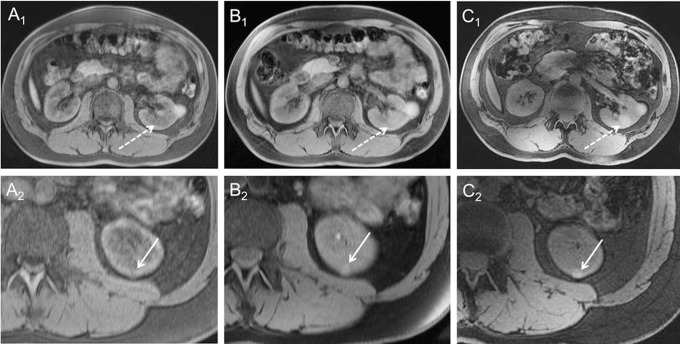
3D VIBE. 3D VIBE imaging at 1.5 Tesla (A), 3 Tesla (B) and 7 Tesla (C) in the same subject. 7 Tesla 3D VIBE imaging demonstrated diagnostic potential by means of detection of pathologies, as it revealed a second hemorrhaged renal cyst (dashed arrow Figure C_1_), not being displayed at lower field strengths. In the second row, arrows show a further very small renal cyst in the same subject, which is also best visible at 7 Tesla (C_2_).

Further increase of the field strength to 7 Tesla showed partially comparable, as well as improved and inferior imaging results with substantial differences for T1 and T2-weighted imaging. Apart from minor restraints due to exacerbated artifact delineation at 7 Tesla, the overall image quality of T1w MRI (2D and 3D) was rated comparably high in all three field strengths. 7 Tesla T1w imaging could be obtained at higher spatial resolution compared to lower field strengths resulting in highest contrast ratio values for analyzed liver, spleen and kidney tissue at 7 Tesla MRI, partly being significant higher compared to 3 Tesla and 1.5 Tesla, with 3 Tesla also showing higher contrast ratio values than 1.5 Tesla for most T1-weighted sequences. Thus, 7 Tesla 3D VIBE imaging allowed for the detection of an incidental hemorrhagic renal cyst, only insufficiently detectable at lower field strengths (Fig 7).

Although being an incidental benign pathology, this finding may demonstrate the diagnostic ability of high spatial resolution imaging at ultra-high magnetic field strength. Our results confirm the previously demonstrated potential of 7 Tesla T1-weighted MRI for non-enhanced angiographic applications, based on an inherently hyperintense signal of the arterial vasculature [36–38]. This feature of 7 Tesla T1w MRI enhances its potential for non-enhanced assessment of abdominal vasculature, particularly in patients with renal insufficiency and dialysis with regards to the association of Gadolinium-based contrast agents and Nephrogenic Systemic Fibrosis [39] as well as recent findings on gadolinium retention in the brain [40].

Along with comparable and beneficial findings, our results also demonstrate current limitations of 7 Tesla abdominal MRI, with a particular focus on residual B_1_ field inhomogeneities. Ultra-high-field imaging in general, and particularly body MRI, is known to be susceptible to RF inhomogeneities due to the shortened RF wavelength at 300 MHz, leading to major signal intensity variations across the imaged FOV. Despite performing successful B_1_ shimming to reduce and focus B_1_ artifacts out of the defined region-of-interest with a custom 8-channel shimming setup, residual inhomogeneities resulted in impairment of overall image quality. Apart from optimization of shim techniques, further optimization of RF pulses may be conducive to improve abdominal MRI at 7 Tesla. Wu et al recently outlined the potential of a multi-spoke slice-selective parallel transmit RF pulse to address B_1_ inhomogeneity in the liver MRI at 7 Tesla, demonstrating improved excitation homogeneity when compared to static B_1_ shimming [41]. Likewise, further optimization of RF coils may be beneficial for improved body imaging at 7 Tesla. Snyder et al. reported on significant advantages in transmit- and receive performance as well as parallel imaging and local / global SAR, when applying a sixteen channel stripline/TEM surface array compared to an eight-channel coil [42]. Nevertheless, the utilization of the eight-channel coil offered eased complexity, construction and handling. Snyder et al. concluded the choice of the applied coil to be determined on an individual basis with regards to RF amplifier configuration, requirements on B_1_ homogeneity and availability of B_1_ optimization methods [42]. Hence, based on experiences in previous trials on 7 Tesla abdominal MRI and to ensure best overall practicality for our trial of three intraindividually performed MR examinations, we decided to utilize an eight-channel coil for this trial.

The combination of the above named residual B_1_ inhomogeneties and SAR restrictions led to a significantly hampered generation of clean refocusing pulses, leading to non-diagnostic T2-weighted TSE images at 7 Tesla with lowest contrast ratio values compared to 3 Tesla and 1.5 Tesla MRI, confirming previously published data [24, 26, 43]. Hence, the assessment of the biliary duct system, in terms of Magnetic Resonance Cholangiopancreatography (MRCP) at 7 Tesla, is not applicable at current status. One attempt to overcome this problem was presented by Fischer et al., investigating the feasibility and diagnostic ability of imaging the biliary duct system applying a biliary secreted Gadoxetic acid (Primovist, Bayer Healthcare) at 7 Tesla in comparison to 3 Tesla MRC [43]. Their initial study results demonstrate the feasibility of contrast-enhanced imaging of the biliary tract at 7 Tesla, enabling equivalent results of the central duct segments compared to 3 Tesla MRCP [43]. Considering a smaller FOV, e.g. MRI of the prostate, B_1_-shimming and refocusing pulses can be performed more effective and T2-weighted imaging at 7 Tesla can achieve diagnostic value [44].

One limitation of our study lies in the lack of the administration of contrast media to perform dynamic imaging. However, the application of a threefold dosage of contrast agent to each healthy subject was prohibited for ethical reasons. Additionally, it has to be mentioned that the examined subjects were young and had a normal BMI, as body height and composition have a strong effect on the B_1_ distribution, particularly at higher field strength. Further investigations, including older and/or larger subjects, patient studies as well as contrast-enhanced data should be the focus of future studies. A further limitation lies in the omission of signal-to-noise (SNR) and contrast-to-noise ratio (CNR) measurements as our study setup was designed to compare clinically standardized examination protocols commonly utilizing parallel imaging, which is known to impede the analysis of absolute SNR and CNR values due to inhomogeneous distribution of signal [31]. Therefore, signal of parenchymatous organs (liver, spleen, left kidney) and corresponding psoas muscle was measured to calculate contrast ratios for T1 and T2w sequences in all field strengths, as previously published in other comparison trials on magnetic field strength [21]. Nevertheless, SNR/CNR analysis is generally feasible by reconstruction of SNR-scaled images [45, 46] and might be the focus of future field strength comparison studies. Lastly, the unavailability of commercial coil concepts for ultra-high-field abdominal imaging enforced the application of a custom-built 8-channel transmit receive coil, certainly impairing the utilization of the full potential of 7 Tesla imaging and a comparison to lower field strengths using commercial receive-only coil systems. The utilization of a transmit-receive coil concept demands for optimization of both modi and compromises, as a transmit-only coil is farther to the body, while the receive-only coil is close to the body to enable higher SNR and Parallel acquisition technique (PAT) factors for parallel imaging. Hence, PAT factors > 2 were not applicable for 7 Tesla imaging, impairing a significant increase in spatial resolution for some of the breath hold sequences.

In conclusion, this intraindividual comparison trial of 1.5 versus 3 versus 7 Tesla in abdominal MRI demonstrates potential benefits and limitations affiliated to the increase of the magnetic field strength. The clinically established increase of the field strength from 1.5 to 3 Tesla offered imaging at increased spatial resolution with comparable image quality and no relevant exacerbation of artifacts. Further increase of the field strength to 7 Tesla demonstrated its high imaging potential, yet also limitations mainly based on the inhomogeneous excitation field compared to lower field strength. Hence, further optimization of dedicated RF coil concepts and RF pulse techniques are expected to better cope with the physical effects associated with ultra-high magnetic field strength.
